# Role of Brown Adipose Tissue in Metabolic Health and Efficacy of Drug Treatment for Obesity

**DOI:** 10.3390/jcm13144151

**Published:** 2024-07-16

**Authors:** Natalia O. Markina, Georgy A. Matveev, German G. Zasypkin, Tatiana I. Golikova, Daria V. Ryzhkova, Yulia A. Kononova, Sergey D. Danilov, Alina Yu. Babenko

**Affiliations:** 1 Laboratory of Prediabetes and Metabolic Disorders, WCRC “Centre for Personalized Medicine”, Almazov National Medical Research Centre, Saint Petersburg 197341, Russiazx5000@mail.ru (G.A.M.); germanzasypkin@yandex.ru (G.G.Z.); tanya.golya@gmail.com (T.I.G.);; 2 Facility of Digital Transformation, ITMO University, Saint Petersburg 197101, Russia

**Keywords:** brown adipose tissue, obesity, metabolic syndrome, weight-reducing therapy, positron emission tomography/computed tomography

## Abstract

(1) **Background**: Brown adipose tissue (BAT) is responsible for non-shivering thermogenesis, and its activation has become a new object as both a determinant of metabolic health and a target for therapy. This study aimed to identify the relationships between the presence of BAT, parameters that characterize metabolic health (glucose, lipids, blood pressure (BP)), and the dynamics of body mass index (BMI) during weight-reducing therapy. (2) **Methods**: The study included 72 patients with obesity. We investigated metabolic parameters, anthropometric parameters, and BP. Dual-energy X-ray absorptiometry (DXA) and positron emission tomography and computed tomography (PET/CT) imaging with ^18^F-fluorodeoxyglucose (^18^F-FDG) were performed. (3) **Results**: Before weight-reducing therapy, BAT was revealed only in 19% patients with obesity. The presence of BAT was associated with a lower risk of metabolic deviations that characterize metabolic syndrome: shorter waist circumference (WC) (*p* = 0.02) and lower levels of glucose (*p* = 0.03) and triglycerides (*p* = 0.03). Thereafter, patients were divided into four groups according to the type of therapy (only lifestyle modification or with Liraglutide or Reduxin or Reduxin Forte). We did not find a relationship between the presence of BAT and response to therapy: percent weight reduction was 10.4% in patients with BAT and 8.5% in patients without BAT (*p* = 0.78) during six months of therapy. But we noted a significant positive correlation between the volume of BAT and the effectiveness of weight loss at 3 months (r = 0.52, *p* = 0.016). The dynamic analysis of BAT after 6 months of therapy showed a significant increase in the volume of cold-induced metabolically active BAT, as determined by PET/CT with ^18^F-FDG in the Liraglutide group (*p* = 0.04) and an increase in the activity of BAT standardized uptake value (SUV mean and SUV max) in the Reduxin (*p* = 0.02; *p* = 0.01, respectively) and Liraglutide groups (*p* = 0.02 in both settings). (4) **Conclusions**: The presence of brown adipose tissue is associated with a lower risk of metabolic abnormalities. In general, our study demonstrated that well-established drugs in the treatment of obesity (Liraglutide and Reduxin) have one more mechanism for implementing their effects. These drugs have the ability to increase the activity of BAT. A significant positive relationship between the total volume of BAT and the percentage of weight loss may further determine the priority mechanism of the weight-reducing effect of these medicaments.

## 1. Introduction

Adipose tissue possesses a set of functions, including endocrine function. Adipocytes are different in terms of their morphology and function. We may divide them into two main types: white adipose tissue (WAT) and brown adipose tissue (BAT). WAT can store energy in the form of triglycerides. At the same time, the excess accumulation of WAT reserves, especially in visceral areas, may increase the relative risk of cardiometabolic disorders [1]. In contrast, BAT may increase the release of energy from glucose and lipid stores through the process of non-shivering thermogenesis to adapt to cold conditions. This ability arises from the presence of numerous mitochondria, along with the high expression of uncoupling protein-1 (UCP1).

There are two types of UCP1-positive thermogenic adipocytes, which arise from distinct developmental lineages [2]. For example, “classical brown adipocytes” derive from cells of the central dermomyotome [3]. Thus, classical brown adipocytes are similar to skeletal muscle in terms of their developmental origin, while brown-like adipocytes are closer to white adipocytes [4]. Such brown-like adipocytes have been named “brite”, “beige”, or “recruitable brown” due to their analogy to white adipocytes and, at the same time, their ability for transformation into recruitable brown adipose tissue.

BAT was considered to have only a thermogenic function, but recent research has shown the endocrine potential of BAT. BAT-derived molecules were discovered and termed “batokines”. They can affect the physiology of a variety of organ systems such as the skeletal muscles, liver, intestine, and central nervous system [5]. It is considered that BAT may improve the oxygenation of cardiomyocytes, increase fatty acid and glucose uptake by skeletal muscles, reduce lipogenesis and inflammation in the liver, and also enhance pancreatic beta cell protection and insulin secretion [6]. Therefore, BAT may have a positive effect on metabolic health, included in the concept of metabolic syndrome (waist circumference (WC), glucose level, triglyceride, and high-density lipoprotein cholesterol levels, blood pressure (BP)). The relationship between BAT and these parameters was also examined as part of our research. Since some criteria include an assessment of the level of C-reactive protein (CRP), we also evaluated this parameter.

Several methods have now been developed to detect and quantify BAT, but positron emission tomography and computed tomography (PET/CT) scanning with ^18^F-fluorodeoxyglucose (^18^F-FDG) remains the “gold standard” for determining the presence of BAT, its activity, and volume in humans. Activated BAT uptakes glucose and ^18^F-FDG [7]. ^18^F-FDG is a labeled glucose analog that is absorbed by metabolically active tissues (heart, central nervous system, and BAT). The radiopharmaceutical ^18^F-FDG has the ability to accumulate not only in tumor cells but also in brown adipose tissue, as noted in publications [8,9]. The results of numerous studies indicate that BAT is activated by cold exposure in the majority of healthy men and women. Furthermore, PET/CT using ^18^F-FDG demonstrates increased uptake of the radiopharmaceutical at sites of active BAT accumulation [7,10,11,12]. This property of BAT easily forms the basis for the use of ^18^F-FDG PET/CT technology after prolonged cold stimulation to study its properties and its physiological significance for human metabolism, including obesity.

Meanwhile, there are many factors that complicate the assessment of BAT by this method, for example, the environmental temperature, season, physical activity, blood glucose levels, and a significant list of nutrients that both increase (coffee, thyme, turmeric, capsanoids, grapes, oolong tea, cocoa, etc.) and decrease (dietary fats) BAT activity. Drugs can also either increase (sympathomimetics, β3-agonists (miramegron), thyroid hormones, catecholamines, fibrates, glitazones) or decrease (beta-blockers, sotalol, statins) BAT activity. This creates the need for strict control and consideration of all these factors when planning studies to evaluate BAT. On the other hand, the presence of the ability to increase BAT activity in drugs has become an additional useful option that increases the attractiveness of these drugs. Glucagon-like peptide-1 (GLP-1) agonists and Sibutramine have also attracted attention as drugs that can increase BAT activity. However, most of the studies that have confirmed the ability to activate BAT for both GLP-1 receptor agonists [13] and Sibutramine were experimental. A human study by Janssen et al. demonstrated that GLP1 receptor agonist (Exenatide) increased the metabolic volume (+28%, *p* < 0.05) and mean standardized uptake value (SUV-mean) (+11%, *p* < 0.05) of cervical and supraclavicular BAT depots in young men without obesity [14]. Meanwhile, Liraglutide differs from exenatide in pharmacokinetics, and pharmacodynamics and may yield excellent results on the effects on BAT. In addition, the obese patient population, which is significantly less likely to be found to have BAT, may show different results on its drug induction. We did not find any studies on the effects of Sibutramine on BAT activity in humans in the available literature, and this is of some interest. Meanwhile, given the fact that weight loss itself can increase BAT volume and activity [15], we included several weight loss interventions in the analysis to be able to clarify the contribution of distinct medications.

Thus, the aim of our study was to assess the relationships between the presence of BAT, parameters of metabolic health, and the influence of BAT on the effectiveness of the therapy with weight-lowering drugs containing Liraglutide and Sibutramine.

## 2. Materials and Methods

### 2.1. Clinical Characteristics of the Examined Patients

All patients signed informed consent prior to enrollment in the study. Inclusion criteria: men, women, age > 18 years, body mass index (BMI) > 30 kg/m^2^, provided there were no significant weight fluctuations (more than 5%) in the previous 3 months before inclusion in the study, BP not higher than 140/90 mm/Hg at the time of inclusion in the study, absence of secondary obesity, and willingness to comply with the recommendations on diet, physical activity, therapy, and preparation conditions for the tests.

Non-inclusion criteria: uncontrolled/poorly controlled arterial hypertension (AH); other diseases that are contraindications to the prescription of one of the drugs used in the study, according to the labels; diseases accompanied by uncontrolled thyroid dysfunction (hypothyroidism or thyrotoxicosis); and taking drugs and supplements that affect browning (sympathomimetics, beta-blockers, mirabegron, resveratrol, etc.) and capsaicin-containing ointments. Based on these criteria, 72 patients (23 males, 49 females) were selected.

The medical histories of the patients were taken (duration of obesity, comorbidity, obesity heredity). Drug history was taken with an emphasis on the absence of drugs and dietary supplements that can affect the activity of BAT (beta-blockers, sympathomimetics, mirabegron, adenosine, fibrates, resveratrol, etc.) In addition, an objective assessment was performed (body weight, BMI, WC), hip circumference/waist circumference ratio, waist circumference/height ratio, resting heart rate (HR), and BP.

Degrees of obesity in patients were determined in relation to BMI according to the WHO classification [16]. BMI is an index of weight-for-height that is commonly used to classify underweight, overweight, and obesity in adults. It is defined as the weight in kilograms divided by the square of the height in meters (kg/m^2^). Thus, the first degree of obesity, according to the BMI classification system, is defined as a BMI of 30.00–34.99 kg/m^2^, the second degree of obesity is a BMI of 35.00–39.99 kg/m^2^, while the third degree of obesity is a BMI of ≥40.00 kg/m^2^.

BP is deemed controlled when it is less than 140/90 mm/Hg in patients younger than the age of 60 years and all ages of hypertensive patients with diabetes and/or chronic kidney disease.

Well-controlled lipid values in our sample mean values that do not lead to cardiovascular risk in relatively young patients without previous cardiovascular events and without the presence of diabetes mellitus (low-density lipoprotein cholesterol (LDL-C) < 3 mmol/L, total cholesterol (TC) < 5.0 mmol/L, high-density lipoprotein cholesterol (HDL-C) > 1.0 mmol/L (male) and >1.2 mmol/L (female), triglycerides (TG) < 1.7 mmol/L). The estimate of insulin resistance (IR) was calculated by a homeostasis model assessment (HOMA) index and ≥4 accepted as IR (HOMA-IR ≥ 4).

### 2.2. Laboratory Methods

All laboratory and instrumental investigations were performed twice before intervention and after 6 months of treatment. On the initial day, patients were required to undergo a series of assessments, including blood tests, anthropometry, BP measurement, and densitometry. On the subsequent day, patients were also expected to present at the clinic in a fasting state for a PET/CT study.

Laboratory examination included determination of the level of fasting glucose, glycated hemoglobin, insulin, total cholesterol, LDL-cholesterol, HDL-cholesterol, triglycerides, and C-reactive protein. Homeostasis model assessment of insulin resistance (HOMA-IR) and Homeostasis Model Assessment of Pancreatic B-cell Function (HOMA-B) were calculated.

Biochemical parameters were assessed using a Cobas c311 automated biochemistry analyzer (Roche, Basel, Switzerland) and commercial kits (Roche reagent kits, Basel, Switzerland). The following are reference values for various biochemical parameters: fasting plasma glucose 3.30–6.10 mmol/L (measurement range 0.11–41.1 mmol/L); total cholesterol (TC) (measurement range 0.1–20.7 mmol/L, normal values 3.50–5.00 mmol/L); serum triglycerides (measurement range 0.1–10.0 mmol/L, normal values < 1.77 mmol/L); high-density lipoprotein cholesterol (HDL-C) (measurement range 0.08–3.12 mmol/L, normal values for females > 1.2 mmol/L, for males > 1.0 mmol/L); and CRP (normal values 0–1 mg/L). Serum insulin levels were measured using a Cobas e411 fully automated immunochemical electrochemiluminescence analyzer (Roche, Basel, Switzerland) and commercial Cobas Insulin Elecsys kits (Roche, Basel, Switzerland) within a measuring range of 1.39–6945 IU/mL and a normal value of 17.8–173.0 pmol/L. The pmol/L to µU/mL conversion factor was 0.144. The HOMA-IR index (insulin resistance index) was used to calculate the formula: HOMA-IR = Glucose mmol L × Insulin µIU mL/22.5, and HOMA-B was calculated using the formula 20 × Insulin µUU/mL/ (Glucose mmol/L-3.5).

### 2.3. Instrumental Methods

Before therapy administration, all patients underwent PET/CT scan with ^18^F-FDG to determine the presence of BAT, its activity, and volume.

The patients included in the study fasted for at least 6 h before ^18^F-FDG injection and only drank warm water during this time. Preparation before this procedure included recommendations to abstain from coffee, capsanoid-containing foods and condiments, and smoking for 48 h. In accordance with the designated dietary guidelines for all patients, the amount of fat in the diet was limited, but before PET/CT, patients were additionally warned to refrain from fatty foods for 24 h. Since there is a sharp seasonal temperature difference in the study region, and it was not possible to conduct both studies (before and after 6 months of treatment) in the same season due to the duration of the study, patients were advised to adhere to isothermal conditions (avoid both being in the cold and hyperthermic conditions (visiting sauna, bath, beach in summer time)) 48 h before the procedure.

PET/CT scan was performed using a scanner Discovery 710, GE. All patients dressed in cotton clothing before starting the cold test. For the cold test, the fixed temperature method was used. Patients were placed in a room with an air temperature of 15 degrees Celsius for 2 h before the start of the PET/CT data acquisition in whole-body mode. After 1 h of cold stimulation, 150–200 MBq of ^18^F-FDG was administered intravenously. PET/CT data acquisition started with a low-dose CT scan (100 mA, 120 kVp) for attenuation correction 1 h after ^18^F-FDG was administrated. The ^18^F-FDG PET whole-body scan immediately followed the low-dose CT scan. The mean effective radiation dose was 5.7 ± 1.3. The areas of ^18^F-FDG hyperaccumulation in the supraclavicular, cervical, paravertebral, mediastinal, and paraaortic regions, which are the most common localization sites of BAT, were included in segmentation.

The SUV represents the value of BAT ^18^F-FDG activation, which was defined as the average and maximal ^18^F-FDG activity in each volume of interest (VOI) [17] divided by the injected dose and corrected to the body lean body mass of each patient. Values, which we measured in patients, included the maximal (SUVmax), average standardized uptake value (SUVmean), and metabolic volume of BAT. The total metabolic volume of BAT was measured using standard software of AW 4.6 work station (GE). The metabolic volume of BAT was measured as the sum of all the voxel volumes within the suspected BAT region where the SUV lean body mass was ≥1.2 and the density by CT was between −190 and −10 HU. This parameter depends on a number of factors. These factors include the following: the source of BAT stimulation (e.g., cold exposure, β-AR agonist, sympathomimetic, overfeeding), the type and intensity of cold exposure applied, cold acclimation status, health status (e.g., healthy, diabetes, obese, after weight loss), and demography (e.g., young vs. older age, men vs. women) [18].

Patients were divided into two categories based on the presence or absence of BAT, as defined by visual analysis of PET images.

Body composition (fat free mass, fat mass, and visceral fat mass) was determined by dual-energy X-ray absorptiometry (DXA) using a GE Lunar Prodigy Advance X-ray densitometer. The GE Lunar DXA scanner provided a special mode for the whole-body scan based on body constitution. For this study, the mode “thick” was used to obtain information about the amount and percentage of fat and lean tissue mass.

Thereafter, patients were distributed to the four groups according to the type of therapy. All patients received similar recommendations for changing nutrition and physical activity. A part of the patients (*n* = 20) received recommendations for changing nutrition and physical activity only (the group of lifestyle modification). The rest of the patients received drug therapy in addition to lifestyle recommendations: 17 patients received Reduxin 10 mg per day, 18 patients received Reduxin Forte (10 mg Reduxin and 850 mg Metformin per day), and 17 patients took Liraglutide (Saxenda) (titration of dose from 0.6 mg to 3.0 mg per day). Patients took therapy for 6 months with their following re-examination.

### 2.4. Statistical Analysis

Statistical analysis was performed using STATISTICA 10 (StatSoft Inc., Tulsa, OK, USA) for Windows. Data were presented as mean ± standard deviation. The distribution of the studied variables deviated from normal (normality test was calculated using the Kolmogorov–Smirnov criterion; dispersion was calculated using the Levene’s test). Due to the fact that the distribution in the groups for some parameters is unequal, the use of simple methods of calculation (Student’s t-criterion, etc.) is inappropriate. Therefore, we used non-parametric statistical methods to evaluate the studied groups (Mann–Whitney test was used to compare two independent samples with an interval scale; Wilcoxon’s t-criterion was used to evaluate the relationship between a factor and a dependent variable to compare two dependent samples with each other by the level of expression of a feature). The power of the study for 45 patients was 0.9. The critical significance level (*p*) for testing statistical hypotheses when comparing statistical indicators was less than 0.05.

## 3. Results

### 3.1. Participants Characteristics

Seventy-two participants (23 men, 49 women) completed the study. The main characteristics of the examined group are presented in Table 1.

Thus, the study group was composed of young patients with an average BMI corresponding to the second degree of obesity, good control of blood pressure and lipid parameters, as well as insulin resistance.

Seventy-two patients underwent PET/CT scanning with ^18^F-FDG to determine the presence of BAT, its activity, and its volume. The characteristics of the patients, as they relate to the presence or absence of BAT, are presented in Table 2.

BAT was positively related to younger age and was detected in 14 patients before starting weight loss therapy. Patients with BAT differed from those without BAT in lower WC scores at comparable BMI.

On studying metabolic parameters, we found that patients with a baseline presence of BAT showed fewer changes in their lipid levels. Specifically, a lower level of TG (*p* = 0.03) was revealed. The fasting glucose level of patients with BAT was not found to be significantly different from that of patients without BAT (*p* = 0.14). As well, patients did not differ significantly in the level of glycated hemoglobin, depending on the presence of BAT (*p* = 0.16).

The incidence of history of impaired glucose tolerance (IGT) was not significantly different between the groups (20.0% without BAT vs. 14.3% with BAT, *p* = 0.43), as was the incidence of arterial hypertension (AH) (23.6% without BAT versus 14.2% with BAT, *p* = 0.46). However, there was a trend towards rarer cases of IGT and AH in patients with BAT. Out of 14 patients with BAT, 2 patients (9.0%) were assigned to the Reduxin group, 5 patients (28%) to the Reduxin Forte group, 2 patients (12%) to the Liraglutide group, and 5 patients (33%) to the lifestyle modification group.

### 3.2. The Effect of BAT Presence on Weight Loss Efficiency and Metabolic Parameters

**Figure 1:**
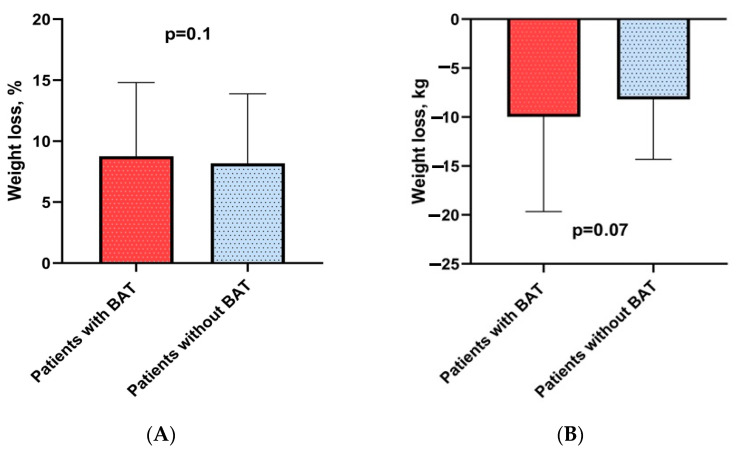
Dynamics of weight loss percentage in patients with and without BAT (**A**). Trend in weight loss in kilograms in patients with BAT versus without BAT (**B**).

There was no statistically significant association between the presence of BAT and response to therapy (weight loss in kilograms and per cent). The average percentage of weight loss was similar in both groups (8.4 ± 6.4% with BAT and 8.3 ± 5.6% without BAT). The difference between groups was not significant (*p* = 0.1) (Figure 1A). Furthermore, no statistically significant differences were identified in weight loss in kilograms based on the presence of BAT. These results are presented in Figure 1B.

Patients with BAT exhibited significantly lower WC compared to patients without BAT (with BAT 94.5 ± 11.4 cm; without BAT 103.9 ± 14.2 cm, *p* = 0.003) before treatment. However, the percentage of reduction in WC between patients with or without BAT was not significantly different after six months of therapy (*p* = 0.13).

After six months of therapy, there was no statistically significant change in fasting plasma glucose levels in either the group of patients with BAT (*p* = 0.07) or the group of patients without BAT (*p* = 0.39).

In the BAT group, TG levels decreased significantly after treatment (*p* = 0.014) in contrast to the group without BAT (*p* = 0.46). Comparative analysis of the characteristics of patients with and without BAT after six months of therapy is presented in Table 3.

**Figure 2:**
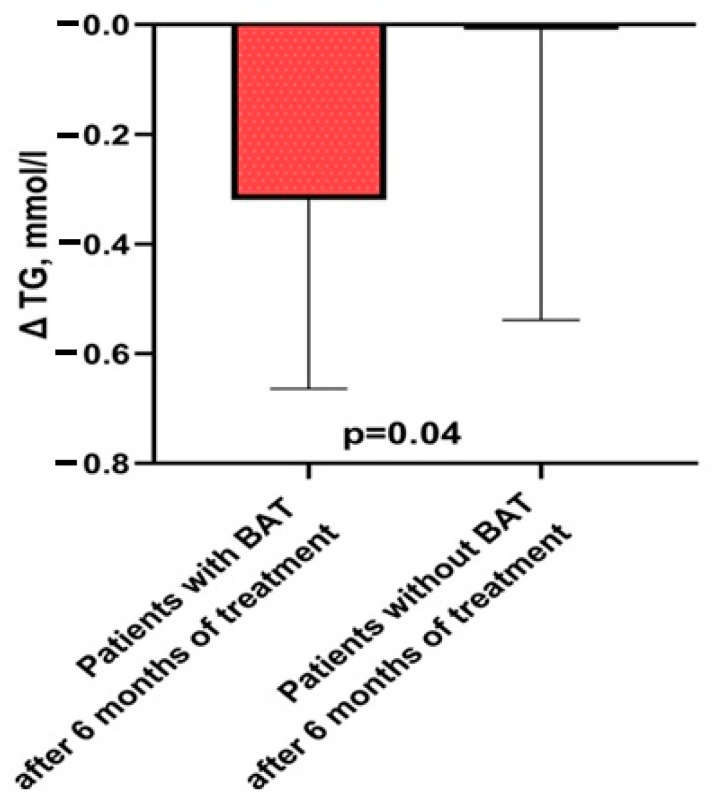
Changes in triglyceride lowering after 6 months of therapy in patients with BAT versus without BAT. Abbreviations: BAT—brown adipose tissue, TG—triglycerides.

The data indicate that significant changes in delta parameters were observed only in the triglyceride parameters (*p* = 0.04) of patients with BAT. These data are presented in Figure 2.

### 3.3. Dynamics of Weight Loss and Metabolic Parameters of Patients with BAT versus without BAT Undergoing Different Types of Therapy

Characteristics of patients who received different treatment options are presented in Table 4. The table shows that patients receiving different treatments did not differ in baseline parameters affecting BAT activity and had baseline differences or differences in dynamics depending on the presence of BAT (age, male/female ratio, BMI, glucose and triglyceride levels, HOMA-IR). Mean weight loss was in the range of 5–10% for all medications with no differences between groups and less than 5% for the group receiving only lifestyle modification recommendations.

Given the limited number of patients with BAT, we were only able to statistically compare weight loss in two groups of patients: those undergoing Reduxin Forte therapy and those engaged in lifestyle modifications.

**Figure 3:**
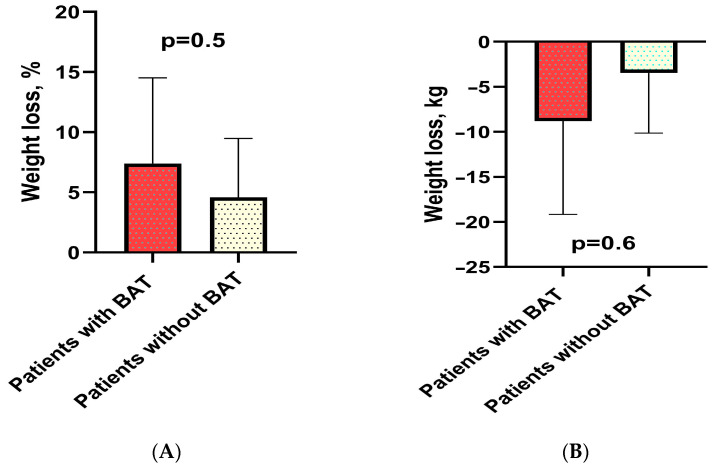
Percentage weight loss in patients with BAT versus without BAT in lifestyle modification group (**A**). Weight loss in kilograms in patients with and without BAT in lifestyle modification group (**B**).

A significant relationship was not identified between the reduction in body weight (percent and kg) in patients with BAT versus patients without BAT in the lifestyle modification group over a six-month period (Figure 3).

**Figure 4:**
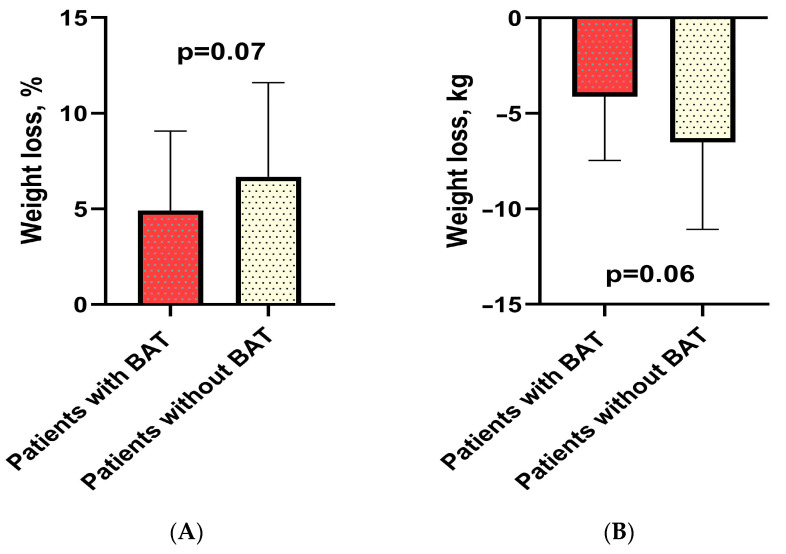
Percentage weight loss in patients with BAT versus without BAT who received Reduxin (**A**). Weight loss in kilograms in patients with and without BAT who received Reduxin (**B**).

Furthermore, no statistically significant differences were observed in body weight loss between patients with and without BAT who took Reduxin Forte. Nevertheless, there was a tendency towards greater efficacy in weight loss among patients with BAT (Figure 4).

### 3.4. Dynamics of Body Composition Parameters according to the Results of Densitometry

We assessed the percentage of body fat by whole-body densitometry. The percentage of total fat loss after therapy also did not depend on the presence of BAT (with BAT 2.65 ± 1.7%; without BAT 2.01 ± 0.8%, *p* = 0.5). The percentage of fat loss from the trunk, which could indirectly reflect a decrease in the visceral type of obesity, likewise did not depend on either the presence or absence of BAT and was approximately 3.06 ± 2.7% in patients without BAT or 2.1 ± 2.15% in patients with BAT (*p* = 0.35).

### 3.5. Changes in BAT during Therapy with Weight-Reducing Drugs

Among patients who had been given Reduxin for six months, the number of those who began to show BAT increased after repeated PET/CT scanning with ^18^F-FDG. Thus, BAT was detected in 9.0% of patients at the baseline. After 6 months of therapy, BAT was detected in 18.2% (*p* = 0.04), while in the other groups, the percentage of patients with indicated BAT did not change significantly (in the Reduxin Forte group *p* = 0.22, in the Liraglutide group *p* = 0.39, in the group of lifestyle modification *p* = 0.5).

A significant increase in the total metabolic volume of BAT was observed in the Liraglutide group over the course of six months of therapy. The initial result was 29.9 ± 43.7 cm^3^, while after six months of therapy, it was 147.2 ± 107.3 cm^3^ (*p* = 0.04).

The index of SUVmax advanced in the Reduxin group: initially, it was 2.71 ± 2.52 g/mL; at 6 months, it was 3.74 ± 1.45 g/mL (*p* = 0.01), and in the Liraglutide group: initially, it was 2.78 ± 3.71 g/mL; at 6 months, it was 8.47 ± 4.46 g/mL (*p* = 0.02). SUV mean also increased significantly in the Reduxin groups: initially, it was 1.79 ± 1.57; after 6 months, it was 1.98 ± 0.84 g/mL (*p* = 0.02), and in the Liraglutide group: initially, it was 1.98 ± 2.36 g/mL; after 6 months, it was 3.52 ± 1.64 g/mL, respectively (*p* = 0.02) (Table 5).

In the lifestyle modification group, there were no significant dynamics in the amount of BAT.

We found a non-significant positive tendency for a relationship between the percentage of body weight loss over 3 months and the total volume of BAT (cm^3^) (r = 0.52, *p* = 0.16). We also identified a tendency for a positive relationship between the percentage of body weight loss over 6 months and the total volume of BAT (r = 0.39, *p* = 0.89).

### 3.6. The Correlation between the Method of Weight-Reducing Therapy and Body Composition Parameters According to Densitometry

Based on the results of densitometry with Reduxin and Reduxin Forte, there was a significant and comparable decrease in total body weight (kg) and total fat percentage. Consequently, the percentage reduction in fat mass in the Reduxin group was 5% (*p* = 0.01), while in the Reduxin Forte group, it was 7% (*p* = 0.001). Despite the implementation of lifestyle modifications, a notable reduction in fat mass was not observed.

## 4. Discussion

Our study is one of the few that have examined not only the relationship between the presence of brown adipose tissue and metabolic health but also the association with response to weight loss interventions. As in other studies [10], we observed a low incidence of BAT in obese individuals. Whereas healthy adults comparable in age to our patients have a reported BAT detection rate of up to 96% [19], in our group of obese patients, BAT was detected in only 19.4.% of patients. The presence of BAT was associated with a lower risk of metabolic abnormalities. Patients with BAT had smaller WC with comparable BMI, which may indirectly indicate less visceral fat, as WC is a surrogate marker of visceral obesity. The change in lipid parameters typical for obesity (triglyceride levels) was more pronounced in patients without BAT.

Our data on the association of the presence of BAT with the studied parameters (younger age, lower BMI, WC, lipid spectrum) mostly coincide with the results obtained by other researchers [7]. For example, the association of BAT reduction with age is explained by ageing, which results in the impaired ability of thermogenic adipocyte precursor cells to proliferate and differentiate, reduced mitochondrial biogenesis, mitochondrial dysfunction [20], and reduced ability to recruit beige adipocytes [21,22,23]. The latter features are viewed as an age-related or obesity-determined “whitening” of thermogenic adipose tissue and are consistent with the reduction in non-shivering thermogenesis [24,25,26,27] and increased risk of cold-induced hypothermia with ageing [28].

We found a lower level of fasting plasma glucose in patients with BAT at the baseline. Data from other researchers on this issue are not always unambiguous. Zhang Q. et al. reported that BAT activity in humans was associated with lower levels of glycated hemoglobin (HbA1c) [29], and a study by Sten van Beek et al. [30] noted that although BAT improves glucose homeostasis in humans, its contribution may be very limited. The exact molecular and physiologic mechanisms of the effect of BAT on the glycemic profile are still unclear [30]. Meanwhile, most researchers tend to believe that BAT has a positive effect on glucose metabolism [7].

We also found no significant differences in the incidence of AH or mean systolic and diastolic blood pressure in patients depending on the presence or absence of BAT at the baseline and after 6 months of therapy. Data from other studies on the relationship of BAT with the level of BP are quite heterogeneous. The browning of perivascular adipose tissue has been found to be negatively correlated with the level of BP. The development of agents that induce the browning of perivascular adipose tissue is a novel strategy for treating obesity-associated AH [31]. The drug-induced browning of subcutaneous fat is also associated with the improved control of BP [32]. However, the role of classical BAT may differ from the role of browning perivascular adipose tissue and subcutaneous fat. BAT produces a lot of biologically active products, such as batokines, which can have effects on BP. Thus, Rouen et al. demonstrated that the increased production of one of the most studied batokines, factor growth fibroblastes-21 (FGF21), counteracts the development of AH and hypertensive remodeling of the heart in the experiments on rats [23]. Other studies on animals have also shown that elevated levels of FGF21 may play a beneficial role in reducing the level of BP.

However, the results of the studies in obese humans differed from the results obtained on rodents’ models of AH. So, it was found that higher serum levels of FGF21 in humans are associated with higher BP [7]. Considering the fact that FGF21 is largely produced by the liver, these data do not uniquely indicate the role of BAT-specific FGF21 in altering BP levels. Also, the ability to modulate (reduce) the level of BP has been demonstrated for other batokine insulin-like growth factor I (IGF-1) [28]. Regarding the role of classical BAT, a large study published in 2021 demonstrated the association of BAT with a lower probability of cardiometabolic diseases, including AH (26.7% in the presence of BAT versus 30.7% without BAT, *p* < 0.0001) in people with an average BMI of about 25 kg/m^2^. When patients were stratified by BMI, it turned out that in patients with BMI > 30 kg m^2^, the differences in the risk of AH were even more pronounced (39.9% in BAT versus 47.8% without BAT, *p* < 0.0001) [33].

The most novel aspect of our study was an attempt to evaluate the possible role of the presence of BAT as a predictor of weight loss in weight loss interventions. We found no significant relationship between the presence of BAT and the percentage of weight loss over 6 months of therapy; we observed a statistically significant decrease in WC and HC both in patients with and without BAT. In addition, based on the results of densitometry, we found no differences in the dynamics of percent total fat mass and percent trunk fat mass depending on the presence of BAT. This may be a consequence of the fact that our study evaluated four different weight loss methods (either lifestyle modification alone or its combination with one of the weight-lowering drugs: Reduxin, Reduxin Forte, and Liraglutide), which could have different effects on BAT activity. The existing literature lacks sufficient data regarding the effects of pharmaceuticals on BAT activity in humans.

Several studies have examined the association of weight loss with BAT activity in obese people influenced by lifestyle modifications [34] or laparoscopic intervention (gastric banding) [35].

The effects of some drugs that have previously demonstrated the ability to increase the amount of BAT in experiments, including β3-adrenoreceptor agonists [36], thyroid hormones [37,38], metformin [39], and thiazolidinediones [40], were tested in terms of their effect on the quantity of BAT in humans, and the results were not always reproducible. The most successful results were for the selective β3-adrenoreceptor agonist Mirabegron, which caused BAT activation and increased metabolic rate in both healthy men [41] and women [42] and obese patients [40]. There was a very moderate loss of body weight and improvement in glycemia and lipid parameters, but BP, on the contrary, increased, as well as heart rate [43].

Metformin, which demonstrated browning in rats [44], showed no effect on the amount of BAT in humans (women with polycystic ovary syndrome [45] and patients with metabolic syndrome combined with human immunodeficiency virus); GLP1 receptor agonists promote weight loss and improve all the cardiometabolic parameters studied by us (glucose, lipids, BP) through several alternative mechanisms. These mechanisms include decreased appetite, changes in gastrointestinal motility, and levels of hormones involved in the regulation of energy balance [46]. According to experimental studies, the reduction in body weight with GLP-1 receptor agonists’ administration is also associated with the stimulation of BAT thermogenesis and adipose tissue browning [47]. Liraglutide stimulates mitochondrial respiration and biogenetics in human adipocytes, potentially through the UCP-1-established browning of adipocytes of BAT [48]. In addition, GLP-1 affects energy homeostasis through the invariant natural killer T (iNKT) located in BAT [49].

We found no studies in the available literature that examined the effect of Liraglutide on the metabolic volume of BAT as measured by PET/CT with ^18^F-FDG in humans. Meanwhile, the research group of LGM Janssen et al. studied the effects on BAT activity and browning of another GLP1 receptor agonists, exenatide [14], and observed a 28% increase in BAT metabolic volume in healthy men localized in the supraclavicular region but did not observe changes in ^18^F-FDG uptake in the subcutaneous adipose tissue (SAT) and visceral adipose tissue (VAT). As noted above, meanwhile, weight loss itself is accompanied by increased energy expenditure and BAT activation [14], making it difficult to assess the contribution of the effects of GLP1 receptor agonists and other weight-lowering drugs to the increase in BAT volume.

Our work has demonstrated the ability of Liraglutide to increase BAT volume and mean and maximal SUV, but we did not find a direct relationship between the presence and amount of BAT and percent body weight and adipose tissue loss. Monoamine reuptake inhibitors such as Sibutramine (Reduxin) induce weight loss by increasing energy expenditure [50]. Regarding Reduxin, we found only experimental studies in the available literature that confirmed the ability of this drug to increase BAT activity [51,52]. This suggests that our study is the first to note the ability of Reduxin to increase both the mean and maximal SUV of BAT.

Meanwhile, our data, on the one hand, showed the ability of Liraglutide and Reduxin to increase the amount of BAT in humans. On the other hand, the lack of association between the severity of weight loss and the presence of BAT indicates that this mechanism is not prioritized for weight loss on these drugs. However, the lack of change in the amount of BAT during treatment with lifestyle modifications alone and with Reduxin Forte in our study and in the works of other authors [34] indicates a role of drugs acting through specific mechanisms in activating BAT. As already mentioned, experimental studies have shown metformin to have an effect on WAT browning [38], while Reduxin increases only BAT activity [52]. Some experimental studies have noted the presence of competitive mechanisms in the activation of BAT and browning [53], which may explain this result. In addition, drawing an analogy with the results obtained in the studies of Miramegron and glitazones, the main effect of BAT activation is not weight loss, which was only slightly decreased on Miramegron and may increase on glitazones. At the same time, these drugs showed significant improvement in metabolic parameters: glucose levels, triglycerides, and insulin sensitivity. Similarly, our study showed the presence of lower glucose and triglyceride levels in patients with BAT before the interventions.

Overall, our study demonstrated that known obesity drugs (Liraglutide and Reduxin) have another mechanism for their effects—the ability to increase BAT activity. In the future, larger studies are needed to obtain more conclusive results regarding the effects of BAT on metabolic health and weight control.

## 5. Limitations of the Study

A strength of our study is the attempt to evaluate the impact of the presence of BAT on the effectiveness of various weight loss interventions. Of note, we evaluated both the effect of lifestyle changes alone versus their combination with weight loss medications. Furthermore, this approach allowed us to differentiate the effect of weight loss itself on changes in the volume and metabolic activity of BAT from the effect of drugs. The presence of drug contribution is indicated by the fact that with comparable weight loss on Liraglutide, Reduxin, and Reduxin Forte, an increase in the volume and activity of BAT was observed on only two of the three studied drugs.

A limitation of our study is that the number of patients receiving different treatments was relatively small. Despite the estimated sample power being met, we had planned to present more patients in each group. Furthermore, we assumed that at least 1/3 of the patients in each group would exhibit BAT prior to the commencement of therapy. However, the difficulty and discomfort associated with performing ^18^F-FDG PET/CT with a cold protocol resulted in a significant loss of patients. Specifically, 43.1% of patients refused to repeat PET/CT, and the percentage of patients who had BAT before treatment was much lower than expected. Nevertheless, the final sample size corresponds to the necessary one according to statistics, and the use of appropriate methods of statistical processing allows us to consider our data correct.

Additionally, a limitation of this study is the lack of randomization. The rationale for this approach is that we did not aim to compare the studied treatments in terms of both efficacy and effect on the metabolic activity of BAT. The objective of this study was to determine whether the presence of BAT affects the percentage of weight loss, and whether the nature of the weight loss intervention affects BAT activity. The lack of randomization was offset by uniform inclusion criteria for all patient groups, which ensured that no significant differences in the parameters studied before the intervention existed. All patients were comparable as if they had been randomized. A significant limitation of this study is the potential influence of numerous factors on BAT activity. These include room temperature during the study, season, and temperature at which the patient was in the period preceding the study (on the way to the study, in the previous 48 h), dietary characteristics, intake of nutrients and medications affecting BAT activity, level of physical activity, age, gender, and comorbidities (e.g., thyroid dysfunction). These issues were addressed as much as possible by detailed inclusion and exclusion criteria, homogeneity of the sample, and detailed pre-test preparation guidelines and examination protocol.

## Figures and Tables

**Table 1:** General characteristics of the included patients.

Characteristic	Patients with Obesity (*n* = 72)
Age, years	37.8 ± 10.9
% of males	31.9
% of smokers	47.2
Weight, kg	105.5 ± 20.5
BMI, kg/m^2^	35.9 ± 4.6
Waist circumference, cm	112.6 ± 14.2
Hip circumference, cm	120.01 ± 10.3
Waist circumference/Hip circumference	0.94 ± 0.11
SBP, mm Hg	124.8 ± 12.5
DBP, mm Hg	74.4 ± 9.1
Glucose, mmol/L	5.4 ± 0.6
Insulin, mmol/L	154.9 ± 100.5
HOMA-IR	5.3 ± 3.6
HOMA-B	275.7 ± 247.4
HbA1c, %	5.43 ± 0.26
TC, mmol/L	4.9 ± 1.05
HDL-C, mmol/L	1.17 ± 0.32
LDL-C, mmol/L	3.0 ± 0.9
VLDL-C, mmol/L	0.71 ± 0.45
TG, mmol/L	1.63 ± 1.03
CRP, mg/L	3.9 ± 3.6

Data are expressed as mean ± SD. Abbreviations: BMI—body mass index. HOMA-IR—homeostasis model assessment of insulin resistance. HOMA-B—homeostasis model assessment–pancreatic b-cell function. SBP—systolic blood pressure, DBP—diastolic blood pressure, HbA1c—glycated hemoglobin, TC—total cholesterol. HDL-C—high-density lipoprotein cholesterol. LDL-C—low-density lipoprotein cholesterol. VLDL-C—very-low-density lipoprotein cholesterol, TG—triglycerides. CRP—C-reactive protein.

**Table 2:** Characteristics of patients depending on the presence or absence of BAT.

Characteristic	Patients with BAT, *n* = 14	Patients without BAT, *n* = 58	*p*-Value
Age, years	30.8 ± 8.5	39.4 ± 10.8	0.006
Weight, kg	103.4 ± 24.9	105.96 ± 19.5	0.31
BMI, kg/m^2^	35.4 ± 4.9	36.05 ± 4.5	0.63
Waist circumference, cm	105.0 ± 16.4	114.4 ± 13.1	0.02
Hip circumference, cm	117.4 ± 10.8	120.7 ± 10.1	0.67
Waist circumference/Hip circumference	0.9 ± 0.13	0.94 ± 0.1	0.09
SBP, mm/Hg	125.7 ± 11.6	124.6 ± 12.8	0.38
DBP, mm/Hg	73.7 ± 9.5	77.4 ± 7.2	0.16
Glucose, mmol/L	5.3 ± 0.45	5.4 ± 0.6	0.14
Insulin, mmol/L	130.6 ± 77.5	161.3 ± 106.0	0.39
HOMA-IR	4.3 ± 2.5	5.6 ± 3.8	0.3
HOMA-B	279.6 ± 302.8	274.7 ± 234.8	0.65
HbA1c, %	5.15 ± 0.45	5.42 ± 0.26	0.16
TC, mmol/L	4.42 ± 0.99	5.00 ± 1.05	0.07
HDL-C, mmol/L	1.17 ± 0.27	1.15 ± 0.23	0.44
LDL-C, mmol/L	2.62 ± 0.78	3.1 ± 0.9	0.052
VLDL-C, mmol/L	0.54 ± 0.2	0.77 ± 0.5	0.1
TG, mmol/L	1.30 ± 0.29	1.69 ± 0.77	0.03
CRP, mg/L	4.71 ± 3.7	3.78 ± 3.6	0.25

Data are expressed as mean ± SD. *p*-values were calculated using Wilcoxon’s signed-rank test. Abbreviations: BAT—brown adipose tissue, BMI—body mass index. HOMA-IR—homeostasis model assessment of insulin resistance. HOMA-B—homeostasis model assessment–pancreatic b-cell function. SBP—systolic blood pressure, DBP—diastolic blood pressure, HbA1c—glycated hemoglobin, TC—total cholesterol. HDL-C—high-density lipoprotein cholesterol. LDL-C—low-density lipoprotein cholesterol. VLDL-C—very-low-density lipoprotein cholesterol, TG—triglycerides. CRP—C-reactive protein.

**Table 3:** Comparison of weight dynamics and metabolic parameters in patients with and without BAT.

Parameter	Patients with BAT before the Treatment	Patients with BAT after 6 Months of Treatment	*p*-Value	Patients without BAT before the Treatment	Patients without BAT after 6 Months of Treatment	*p*-Value
Waist circumference, cm	105.0 ± 16.4	95.1 ± 11.2	<0.01	114.4 ± 13.2	103.6 ± 13.1	<0.001
Hip circumference, cm	117.3 ± 10.7	111.7 ± 10.9	0.02	120.6 ± 10.2	115.3 ± 12.6	<0.001
TG, mmol/L	1.16 ± 0.36	0.85 ± 0.3	0.04	1.69 ± 1.03	1.65 ± 0.95	0.46
Glucose, mmol/L	5.67 ± 0.44	5.4 ± 0.49	0.07	5.28 ± 0.65	5.18 ± 0.69	0.309

Data are expressed as mean ± SD. *p*-values were calculated using Wilcoxon’s signed-rank test. Abbreviations: BAT—brown adipose tissue, TG—triglycerides.

**Table 4:** Baseline characteristics of patients allocated to different weight-lowering therapy options.

Characteristic	Reduxin (1)	Reduxin Forte (2)	Liraglutide (3)	Diet (4)	*p* 1 vs. 2	*p* 1 vs. 3	*p* 1 vs. 4	*p* 2 vs. 3	*p* 2 vs. 4	*p* 3 vs. 4
Age, years	37.2 ± 8.4	35.8 ± 11.3	42.3 ± 13.2	35.8 ± 10.6	0.5	0.2	0.7	0.1	0.9	0.2
Male/female (%)	45/55	16/84	27/73	40/60	0.07	0.2	0.7	0.4	0.1	0.4
Δ Weight, kg	−10.0 ± 5.83	−6.89 ± 5.48	−10.47 ± 4.84	−3.45 ± 6.67	0.2	0.6	0.09	0.01	0.2	0.06
Δ Weight, %	−9.56 ± 6.18	−7.42 ± 5.89	9.85 ± 4.1	4.59 ± 4.87	0.4	0.5	0.09	0.5	0.2	0.07
BMI, kg/m^2^	36.81 ± 4.4	34.89 ± 4.42	36.5 ± 4.75	35.3 ± 4.8	0.1	0.8	0.3	0.3	0.8	0.4
Glucose, mmol/L	5.18 ± 0.64	5.44 ± 0.46	5.46 ± 0.7	5.4 ± 0.6	0.1	0.1	0.1	0.9	0.8	0.9
Insulin, mmol/L	173.1 ± 108.8	143.9 ± 128.3	177.2 ± 86.8	119.2 ± 52.8	0.1	0.6	0.1	0.06	0.6	0.08
HOMA-IR	5.72 ± 3.69	5.1 ± 4.65	6.18 ± 3.09	4.13 ± 1.89	0.2	0.5	0.2	0.08	0.7	0.09
TC, mmol/L	4.88 ± 1.12	4.47 ± 0.98	5.28 ± 1.03	5.01 ± 0.95	0.2	0.3	0.5	0.04	0.1	0.3
HDL-C, mmol/L	1.08 ± 0.3	1.24 ± 0.27	1.22 ± 0.4	1.15 ± 0.3	0.08	0.3	0.4	0.6	0.2	0.7
LDL-C, mmol/L	2.97 ± 0.84	2.67 ± 0.84	3.42 ± 0.93	3.03 ± 0.94	0.2	0.1	0.9	0.03	0.2	0.1
TG, mmol/L	1.81 ± 1.18	1.2 ± 0.5	1.7 ± 0.97	1.79 ± 1.25	0.08	0.9	0.6	0.08	0.1	0.9
CRP, mg/L	3.75 ± 2.78	5.0 ± 5.1	3.37 ± 3.1	3.63 ± 3.29	0.7	0.6	0.7	0.36	0.5	0.8

Data are expressed as mean ± SD. *p*-values were calculated using Wilcoxon’s signed-rank test. Abbreviations: BMI—body mass index. HOMA-IR—homeostasis model assessment of insulin resistance. TC—total cholesterol. HDL-C—high-density lipoprotein cholesterol. LDL-C—low-density lipoprotein cholesterol, TG—triglycerides. CRP—C-reactive protein.

**Table 5:** Quantitative characteristics of BAT in patients before and after treatment.

	Reduxin		
Characteristic	0 months of therapy	6 months of therapy	*p*-value
Total volume of BAT (cm^3^)	6.19 ± 5.54	30.97 ± 33.96	0.08
SUV max (g/mL)	2.71 ± 2.52	3.74 ± 1.45	0.01
SUV mean (g/mL)	1.79 ± 1.57	1.98 ± 0.84	0.02
	Reduxin Forte		
	0 months of therapy	6 months of therapy	*p*-value
Total volume of BAT (cm^3^)	77.0 ± 123.1	128.0 ± 199.2	0.98
SUV max (g/mL)	1.77 ± 1.75	3.83 ± 3.91	0.78
SUV mean (g/mL)	0.97 ± 0.87	1.66 ± 1.28	0.82
	Liraglutide		
Characteristic	0 months of therapy	6 months of therapy	*p*-value
Total volume of BAT (cm^3^)	29.9 ± 43.7	147.2 ± 107.3	0.04
SUV max (g/mL)	2.78 ± 3.71	8.47 ± 4.46	0.02
SUV mean (g/mL)	198 ± 2.36	3.52 ± 1.64	0.02
	Lifestyle modification		
Characteristic	0 months of therapy	6 months of therapy	*p*-value
Total volume of BAT (cm^3^)	208.6 ± 239.8	404.5 ± 384.5	0.1
SUV max (g/mL)	9.9 ± 5.46	13.1 ± 4.1	0.6
SUV mean (g/mL)	3.46 ± 1.6	4.1 ± 1.2	0.6

Data are expressed as mean ± SD. *p*-values were calculated using Wilcoxon’s signed-rank test. Abbreviations: SUV max—maximal standardized uptake value; SUV mean—mean standardized uptake value.

## Data Availability

Data can be provided upon request.
